# St. Corona – eine Fürsprecherin gegen Seuchen? Eine Miszelle aus der Medizin- und Urologiegeschichte

**DOI:** 10.1007/s00120-020-01209-6

**Published:** 2020-05-04

**Authors:** Friedrich H. Moll, Marie-Isabelle Schwarzburger

**Affiliations:** 1 Institut für Geschichte, Theorie und Ethik der Medizin, Heinrich-Heine-Universität, Düsseldorf, Deutschland; 2 Urologische Klinik, urologischer Arbeitsplatz Krankenhaus Merheim, Kliniken der Stadt Köln gGmbH, Köln, Deutschland; 3 Curator Museum, Bibliothek und Archiv zur Geschichte der Urologie, Deutsche Gesellschaft für Urologie e. V., Berlin-Düsseldorf, Berlin, Deutschland; 4 Institut für Geschichtswissenschaften I, Mittelalterliche Geschichte, Heinrich-Heine-Universität, Düsseldorf, Deutschland

**Keywords:** Hl. Corona, Hagiographie in Medizin und Urologie, Erinnerungskultur, Volkskunde, St. Corona, Hagiography in medicine and urology, Culture of remembrance, Folklore

## Abstract

Hagiographie spielt in der Wissenschaftsgeschichte noch immer, gerade bei lokalgeschichtlichen Analysen, eine wichtige Rolle. Während sich in der Urologie das Wissen um den Hl. Liborius gehalten hat, ist die Bekanntheit der Seuchenheiligen insbesondere denjenigen, die bei Geschlechtskrankheiten angerufen werden, in der Regel gesunken.

## Abstract (en)

The knowledge of hagiography and hagiotherapy still plays an important role in the history of science, especially when focusing on specific aspects of history. While knowledge about St. Liborius persists in urology, knowledge about patron saints for pandemics, especially those who were called upon to treat venereal diseases, has diminished due to the association with nonappropriate sexual behavior.

## Einleitung

In der fachkulturellen Erinnerung der Urologie sind bei Harnsteinleiden neben der Heiligenvita [1, 2] des Hl. Liborius [3, 4] in der Regel die des Hl. Apollinaris im Rheinland oder des Hl. Rasso [5] in Bayern bekannt, manchmal wird noch der Hl. Benedikt zitiert [6].

Für den Bereich der Seuchen und Geschlechtskrankheiten („Widerfahrnis“) ist zumeist der Hl. Georg, der Hl. Pellegrinus, der Hl. Dionysius oder besonders im Rheinland der Hl. Apollinaris von Ravenna oder der Hl. Rochus von Montpellier noch in der Tradition und als Stationsbezeichnung in Krankenhäusern in Gebrauch [7–10].

Gegen Impotenz bzw. Unfruchtbarkeit und Kinderlosigkeit/für Kindersegen werden je nach Region eine ganze Anzahl von Heiligen u. a. Hl. Anna, Hl. Andreas, Hl. Antonius von Padua („Ferkels“ oder „Schlüssels Tünn“ in Köln), Hl. Margarete von Antiochien (letztere zählt zu den vierzehn Nothelfern) und Hl. Verena verehrt, bei denen oftmals noch aus vorchristlicher Zeit stammende Riten, wie in der Kirche Santa Maria Francesca delle Cinque Piaghe in Neapel ein Sessel, eine Rolle spielen. Auf diesen setzen sich die Frauen mit unerfülltem Kinderwunsch und bitten bei der ehemaligen Besitzerin des Stuhls, der Hl. Maria Franziska, um eine Schwangerschaft [11]. Ein weiterer ähnlicher Ritus ist für den Hl. Hypatius von Gangra^1^ dokumentiert [12].

Vom Mittelalter bis in die Neuzeit war es üblich, bei gesundheitlichen Beschwerden und/oder Einschränkungen bei den Heiligen der christlichen Kirche um Hilfe zu bitten, denn die Heiligen galten als direkte Verbindung zu Gott [13, 14]. Diese Fürbitten konnten durch ein Gebet, eine Opfergabe oder eine Wallfahrt geschehen, aber auch durch das Aufstellen eines Bildnisses und durch den Erwerb einer Reliquie. Hierbei ist es wichtig zu erwähnen, dass in einigen Fällen eine feste Absichtserklärung für eine Heilung schon reichte. Jeder Heilige war Patron für eine bestimmte Krankheit oder ein Leiden [15]. Darüber hinaus waren und sind Heilige Patrone von Städten und Berufsgruppen (so waren Caesarius von Nazianz, die Hl. Cosmas und Damian oder der Hl. Panthaleon Schutzheiligen der Ärzte). Bei widernatürlichen Gelüsten („griechische Liebe“) wurde in Italien zum Beispiel der Hl. Bernadino oder der Hl. Isidor angerufen [16].

Das Wissen über Hagiotherapie, also die Anrufung von Schutzheiligen für das Kurieren einer Krankheit [17], wird heute kaum noch weitergegeben und einem breiten Publikum zur Verfügung gestellt. Zwar verfügt jede Kirche über Heiligenbildnisse und -altäre und damit die Möglichkeit, Fürbitte zu leisten, jedoch schwindet nach und nach die Bekanntheit der einzelnen Schutzpatrone. So ist das volkskundliche Wissen wie „Gebete bei Steinkolik“^2^ oder magische Handlungsanweisungen bei Steinen oder Pollutionen^3^^,^^4^^,^^5^ in der Regel verlorengegangen [4, 18, 19].

## Heilige Corona (Stefan[i]a) – S Corona matrona Martyr. in Aegyt.

Zu Beginn des Jahres 2020 beherrscht das sog. „Coronavirus“ die Schlagzeilen, auch der urologischen Fachpresse [20]. Benannt ist diese Art von Virus nach dem lateinischen Begriff corona „Krone“, da es eine Art Krone oder Strahlenkranz umgibt [21]. Das Covid-19-Virus gehört zu dieser Virusgruppe.

Die Hl. Corona verbindet mit dem zurzeit pandemischen Covid-19-Virus außer dem Namen noch mehr: Sie gilt in wenigen regionalen Bezügen als Schutzpatronin gegen Seuchen [22]^6^, aber auch in Gelddingen (Österr. Münzeinheit bis 1924 „Krone“) und der Lotterie. Sie ist Patronin der Berufsgruppe der Metzger (Wortähnlichkeit caro lat: Fleisch). Als Reliquien verehrte Überreste von ihr finden sich in Aachen. Der mehr als 100 Jahre alte Schrein wird gerade restauriert und soll spätestens im Sommer in der Aachener Schatzkammer gezeigt werden [26]. Der katholische Gedenktag ist der 20. Februar/14. Mai, der orthodoxe der 11. November [25, 27].

Eine Krone war in der griechischen und römischen Antike ein zu kultischen Zwecken getragener Kranz aus Blumen, Blättern oder Zweigen bzw. die Nachbildung eines solchen Kranzes aus Metall. Demeter oder Kore werden so dargestellt. Der Palmzweig ist ein Friedenssymbol.

Auch der aus dem Griechischen στέφανος analog gebildete Name „Stephana“ bedeutet Bekränzung, Kranz als Ehrenkranz, Kranz beim Opfer für die Götter und auch der Kranz, der im Kampf getragen wurde. Später wurde auch die Kopfbedeckung der Bischöfe der Ostkirche als Stephanos bzw. Mitra bezeichnet.

Laut Legende, die meistens auf die Acta Sanctorum [28] oder das Martyrologium Romanum [29] zurückgehen, soll Corona, in der Regel als Ehefrau des Hl. Viktor beschrieben, etwa 16 Jahre alt gewesen sein, als sie vor rund 1800 Jahren den frühchristlichen Märtyrertod um ca. 177? oder laut anderen Quellen um 303? starb, entweder unter Antonius Pius (Kaiser, 138–161) oder Gaius Aurelius Valerius Diocletianus, genannt Diokletian (Kaiser, 284–305). Ein römischer Statthalter habe die junge Christin mit Seilen zwischen zwei herabgebogene Palmen spannen lassen – durch das Zurückschnellen sei ihr Leib in Stücke gerissen worden. Diese Hinrichtungsart mit Zerren an den Gelenken und Abtrennung vom Körper war in Form der Vierteilung noch bis in das Mittelalter bekannt [30]. Es sind mehrere Orte des Martyriums von Damaskus, Antiochia (koptische Quellen), Alexandria bis Marseille bekannt [23, 28, 31–33].

In Syria sanctorum Martyrum Victoris et Coronae, sub Antonino Imperatore; ex quibus Victor a Sebastiano Judice variis et horrendis affectus est cruciatibus. Cum autem ipsum Corona, uxor cujusdam militis, coepisset beatum praedicare ob martyrii constantiam, vidit duas coronas de caelo lapsas, unam Victori et alteram sibi missam; cumque hoc audientibus cunctis testaretur, ipsa quidem inter arbores scissa, Victor vero decollatus est [29].

Der Name „Corona“, lateinisch „die Gekrönte“, weist ebenso wie der griechische Name „Stephana“ von Stephanus auf den allgemeinen Begriff „Märtyrerin“ hin.

Da beide Heilige durch ihre Legende eng miteinander verbunden sind, wird die Vita der Hl. Corona häufig mit der des Hl. Viktor, ihrem Ehemann (oder einem Kamerad ihres Mannes), zusammen dargestellt. Die Festtage gelten auch für beide Heilige. Eine Verehrung in Nord- und Mittelitalien ist bereits für das 6. Jahrhundert belegt.

## Reliquien

Reliquien (von lateinisch „reliquiae“ – Zurückgelassenes, Überbleibsel) sind Gegenstände religiöser Verehrung. Besonders Körperteile oder Teile des persönlichen Besitzes eines Menschen, der von der katholischen Kirche als Heiliger verehrt wird, gehören hierzu [34]. Eine Reliquienverehrung ist Bestandteil katholischer Volksfrömmigkeit. In der evangelischen Kirche ist dieser Brauch unbekannt. Für den Reformator Martin Luther (1483–1546) waren Reliquien nicht heilig: „Es ist alles tot Ding, das niemand heiligen kann“ [35]. Reliquien brachten im Mittelalter Macht, Ansehen, Pilger und Spenden. Das schuf eine gewisse Nachfrage nach ihnen. Und diese Nachfrage schuf wiederum ein Angebot [34].

Reliquien werden allgemein in drei Kategorien eingeteilt, die auch ihre Hierarchisierung wiederspiegeln. So sind unmittelbare Reliquien alle Körperteile der Heiligen. Dies können u. a. Knochen und Haare sein. (Reliquien erster Klasse). Zu den mittelbaren Reliquien oder echten Berührungsreliquien, gehören Gegenstände, die von den Heiligen noch zu ihren Lebzeiten berührt wurden. Die sog. „künstlichen“ sind jene, die echte Reliquien berührt haben [36].

Diese Kategorisierungen spiegeln nicht nur die Bedeutung der Reliquien selber, sondern auch die der besitzenden Institution wieder. So waren natürlich Reliquien der ersten Klasse neben den Christusreliquien besonders begehrt.

## Reliquientranslation

Kaiser Otto III. (980–1002, Kaiser ab 996), der ebenfalls im Aachener Dom bestattet wurde, soll im Jahr 997 n. Chr. die Überreste der Hl. Corona und des Hl. Leopardus von Otricoli/Umbrien nach Aachen gebracht und in der Aachener Münsterkirche beigesetzt haben. Seither gelten beide Heilige als Mitpatrone des Aachener Marienstifts [37].

Eine andere Version lässt es möglich erscheinen, dass bereits Karl der Große (747/48–814, Kaiser ab 800) die Gebeine der Hl. Corona und des Hl. Leopardus nach Aachen überführen ließ.

Wann Corona in einem Kanonisationsprozess heiliggesprochen wurde, ist nicht bekannt. Es lassen sich aber durch Patrozinien Rückschlüsse ziehen. Da Leopardus und Corona zusammen als Conpatrone zur Weihe des Aachener Marienstiftes Ende des 8. Jahrhunderts benannt wurden, lässt darauf schließen, dass die Heiligsprechung vor dem Jahr 800 erfolgt sein muss.

Die Grabplatten sind bis heute im Aachener Dom zu sehen. 1843 – bei Grabungen auf der Suche nach dem Bestattungsort Karls des Großen [38] sowie 1910 wurden die Reliquiensärge aus Blei sowie die Gebeine ausgegraben und ab 1911/1912 in Schreine gelegt [39, 40]. Die Reliquiensärge werden in der Michaelskapelle des Aachener Doms aufbewahrt [41–44].

Kaiser Karl IV. (1346–1378, Regierung 1346–1378) brachte weitere Reliquien der Hl. Corona aus Feltre in den Prager Dom. Hierfür wird eine Rolle gespielt haben, dass man sich in Prag bemühte, Reliquien mit Namen von Heiligen, die Sieges- oder Triumphnamen besaßen oder daran anklangen, zu erwerben [45].

Weiter befinden sich Reliquien seit 965 n. Chr. im Dom zu Bremen. Hier wird die Hl. Corona viel verehrt, so finden sich Abbildungen von Ihr auf dem Chorgestühl des Bremer Doms und auf der Westempore [46]. In Bremen wurden auch Pilgerzeichen im Gitterguss mit Corona Motiv gefunden [47, 48].

Weitere Reliquien finden sich in Italien als frühen Ort der Verehrung beispielsweise in Castelfidardo bei Osimo an der Adriaküste bei Ancona oder in Otricoli/Umbrien s. oben (Santa Maria Assunta), wo auch der Hl. Leopardus verehrt wird [49, 50].

Dass es viele verschiedene Aufbewahrungsorte der Reliquien gibt, ist daher möglich, da oft schon kleinste Knochenfragmente in den Reliquiaren aufbewahrt werden. Weiter können einige auch aus der letzten Kategorie der Reliquien stammen. Diese lässt die Anzahl an möglichen Reliquien exponentiell steigen.

## Patrozinien

In Österreich sind Patrozinien in St. Corona am Wechsel (NÖ) sowie St. Corona am Schöpfl (Gemeinde Altenmarkt) bekannt. Ebenfalls gibt es ein Patrozinium St. Corona in Handlab bei Passau sowie in Arget, Gemeinde Sauerlach, Obb., sowie Unterzarnham (Kreis Mühldorf am Inn).

Im Bistum Regensburg sind sogar drei Patrozinien der heiligen Corona verzeichnet: die ehemalige Wallfahrtskirche St. Corona in Altenkirchen bei Frontenhausen, die Pfarrkirche St. Corona in Staudach bei Eggenfelden sowie die Nebenkirche Koppenwall in der Pfarrei Pfeffenhausen bei Landshut. Wahrscheinlich war der Corona-Kult über Böhmen und Niederösterreich auch ins südöstliche Bayern gekommen [51–53].

In Italien besteht ein Patrozinium in Vicenza, in Frankreich zusammen mit dem Hl. Viktor in Ennezat (Département Puy-de-Dôme) sowie in Italien in Grazzano Badoglio (Provinz Asti) und Feltre.

### Infobox

*Andachtsbilder, Heiligenbilder, Fleißkärtchen* mit religiösen Motiven wurden mit Entwicklung der Fotographie, der Chromolithographie im 19. Jahrhundert sowie des Mehrfarbenrasterdruckes im 20. Jahrhundert bei Benutzung von Schnellpresse zu Massenwaren. Als Wandbilddruck besonders mit „Schutzengelbildern“ fanden religiösen Motive vermehrt Eingang in bürgerliche Wohnräume. In dieses Genre im Stil der Nazarener fielen auf nichtreligiöser Ebene der mythischen „Elfenreigen“ oder der „Röhrende Hirsch“, Motive, die viele Verlagshäuser im Programm hatten. Erst in den letzten Jahren wurde dieser Teil der Populärkultur wissenschaftliches Untersuchungsgebiet. Noch heute sind Angebote besonders in den romanischen Ländern und in Südamerika im Handel, meist mit religiösen Motiven.

## Volkskundliche Aspekte des Corona-Kults

Das Corona-Gebet (Kronengebet) ist ein volksmagisches Ritual, besonders in Niederösterreich und Böhmen, das während des 17. und 18. Jahrhunderts populär war. Es sollte zum Aufspüren verborgener Schätze dienen und wurde mehrfach erwähnt [54–56]. Gerichtsprozesse in der frühen Neuzeit, die sich mit magischer Schatzsuche befassten, siedelten das Delikt meist nicht im Bereich der Magie an, sondern werteten es als Betrug. Die Verbindung der Heiligenlegende hiermit blieb in der Forschung unklar, wahrscheinlich ist es auf die wortgleiche Münzbezeichnung „Krone“ zurückgeführt [57].

Gebete an die Hl. Corona lassen sich bis in das 20. Jahrhundert nachweisen [58, 59].

## Darstellungen der Hl. Corona

In der christlichen Kunst werden Heilige mit Attributen dargestellt, um diese kenntlich zu machen oder das Martyrium zu charakterisieren. Neben der Kleidung sind das oft Gegenstände oder beigeordnete Tiere [60, 61].

Die Hl. Corona wird in der Kunst zumeist als junge Frau dargestellt, da sie mit 16 Jahren gestorben ist. Sie ist in den Darstellungen als Märtyrerin durch die Attribute Krone und Palmzweig zu erkennen oder anhand der Darstellung ihres Martyriums. Dieses zeigt sie, nach ihrer Legende, zwischen zwei über Kreuz gespannten Palmen. In vielen dieser Abbildung sind auch zwei römische Soldaten zu sehen, welche die Palmen gespannt halten.

### Darstellung als Märtyrerin

**Figure 1:**
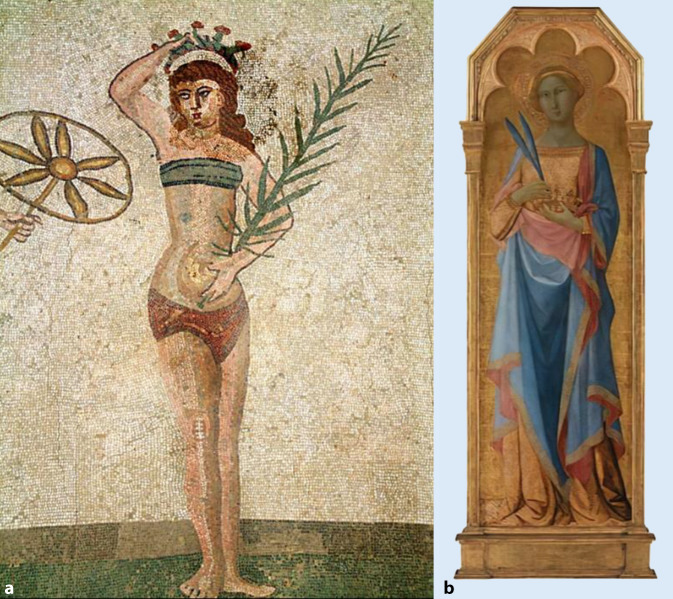

Auf einem Altarbild wird die Hl. Corona nach römischen Vorbild (Abb. 1a) mit Palmzweigen, die auf ihr Martyrium hindeuten und einer Krone dargestellt. Die kreisförmige Aureole ist nur schwer zu erkennen. Schon bei den Römern wurden Herrscher mit einem Nimbus (lat. Wolke) dargestellt [62].

### Darstellung des Martyriums (Abb. 2)

### Andachtsbilder mit dem Martyrium der Hl. Corona als Massenware

**Figure 2:**
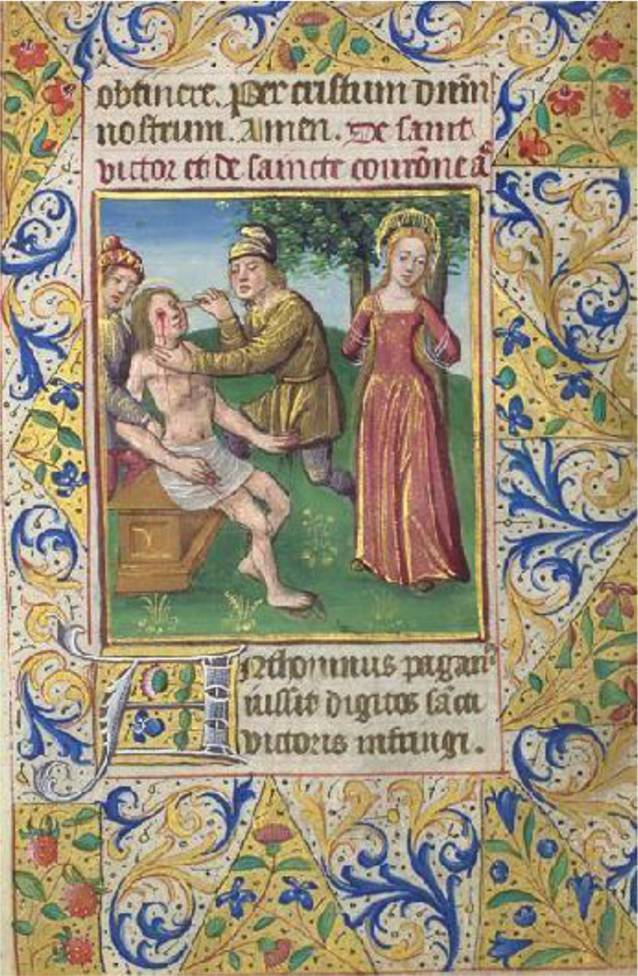

**Figure 3:**
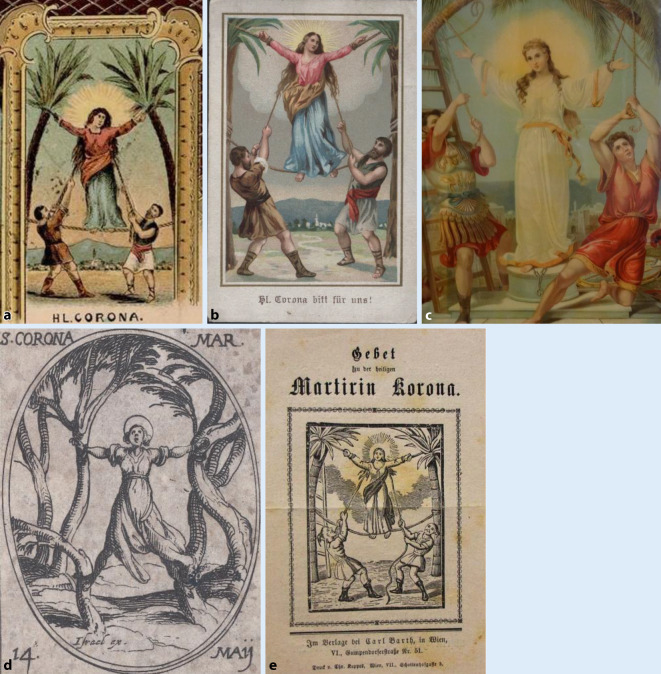

Die Andachtsbilder mit dem Martyrium der Hl. Corona (Abb. 3a–c) sind von der Darstellung her zunächst sehr ähnlich. Abgebildet sind zwei römischen Soldaten, die die beiden Palmen gespannt halten, zwischen denen Corona gespannt wird. Auch hier ist sie eine junge Frau. Der Nimbus im Hintergrund erhebt sie, auch durch die fast schon schwebende Darstellung, in den Stand der Heiligen. Das Bildmotiv mit schwebender Corona ist schon von Jacques Collot (1592–1635) verwandt worden ([63]; Abb. 3d) In der Abbildung aus einem Stundenbuch eines unbekannten Autors um 1480 (Abb. 2) steht die Heilige noch zwischen Bäumen, die nicht als Palmen zu erkennen sind.

Das Bildmotiv der Marter eignet sich durch seine ausdrucksstarke Formensprache auch als schwarz-weiße Abbildung (Abb. 3e).

**Figure 4:**
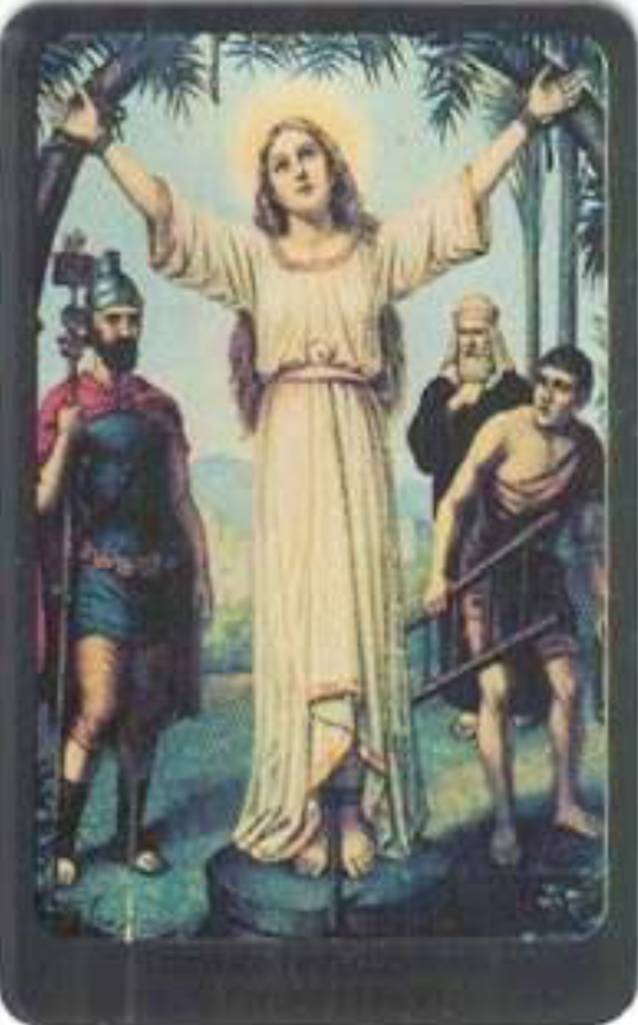

Der Künstler Friedolin Leiber (1843–1912), der in Frankfurt für das Verlagshaus Edward Gustav May arbeitete und der für seine „Schutzengelbilder“ bekannt wurde, nutze ebenfalls das Motiv des Martyrium der Hl. Corona (Abb. 4). Die Heilige wir von ihm mit einem weißem Kleid als Farbe der Unschuld und Reinheit dargestellt. Ansonsten überwiegen Darstellungen der Heiligen mit Kleidern in rot-blauer Farbe (Rot für Martyrium und Glaube, Blau für Himmel). Leibers Abbildung ist an die älteren Motive aus Österreich (St. Corona am Wechsel) deutlich angelehnt. Er lässt aber den Ortsbezug in der Landschaftsdarstellung im Hintergrund bewusst aus, wahrscheinlich, um die Verkaufsmöglichkeiten ohne einen expliziten lokalen Bezug zu steigern [64, 65]. Noch heute wird diese Abbildung über ein online Versandhaus vertrieben [66].

**Figure 5:**
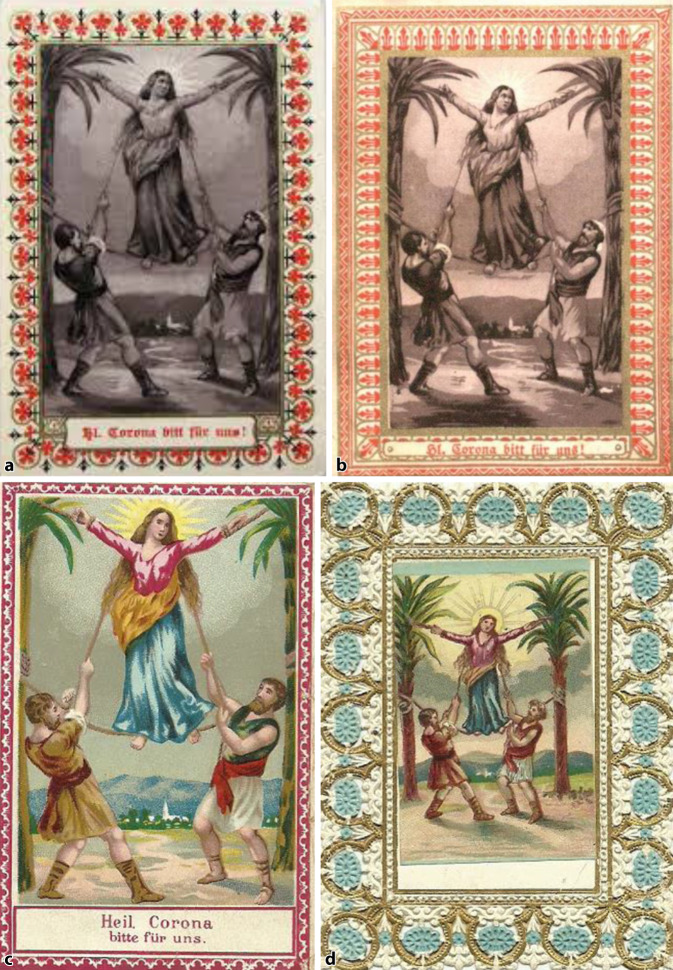

Die Corona-Andachtsabbildungen aus Österreich (Abb. 5a–d sowie 6a–e) aus St. Corona am Wechsel wurden als Massenwaren an vielfältige Anforderungen des populären Geschmacks der Zeit und der Preisgestaltung angeglichen, nicht nur in der Farbgebung (Abb. 5a–c). Bei einfarbigen Abbildungen wurde der Rand in besonderen Farben betont. Auch wurden verschiedene kurze Fürbitten oder Gebete eingedruckt. Die Blumenumrandungen waren teils nur aufgedruckt (Abb. 5a–c) oder auch aufwendiger gestanzt bzw. geprägt und die Marterdarstellung als Chromolithographie eingeklebt (Abb. 5e, f; [67, 68]).

### Darstellungen als Schutzpatronin einer Kirche

**Figure 6:**
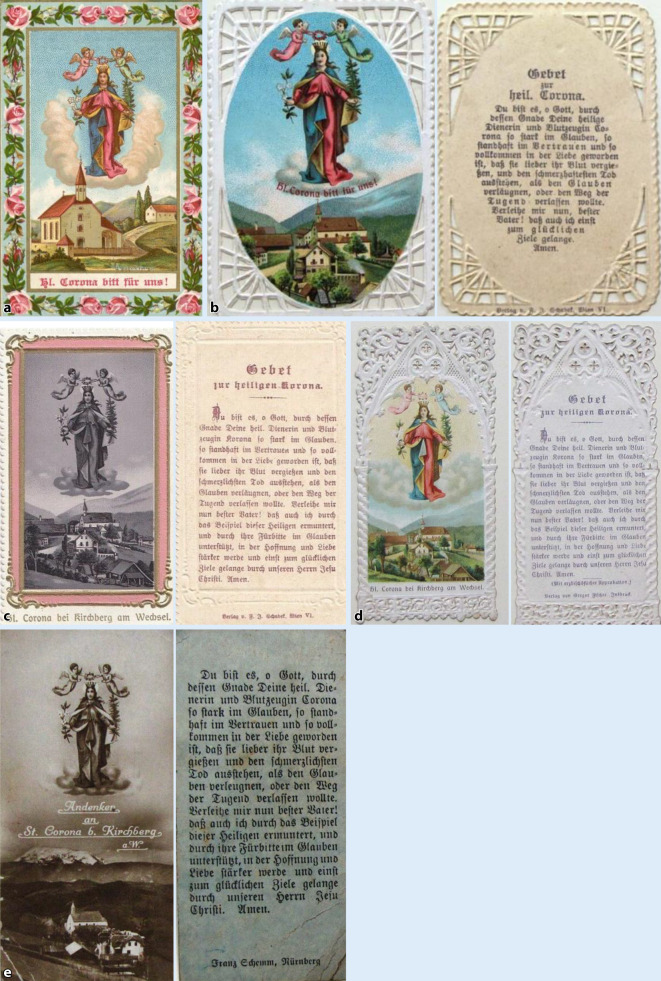

In den folgenden Abbildungen ist die Hl. Corona als Patronin der ihr geweihten Kirchen dargestellt. Anders als in den vorherigen Andachtsbildern, sieht man sie hier über der Kirche schwebend. Sie ist förmlich in den Himmel aufgestiegen, die zwei schwebenden Engel krönen sie mit einer Blätterkrone. Dieses Bildmotiv ist an zeitgenössische Mariendarstellungen unverkennbar angelehnt ([69]; Abb. 6a–c).

Bei der Wallfahrtsandachtsabbildung (St. Corona am Wechsel) des Wiener Verlags Schabek (5,4 cm × 7,8 cm; Abb. 6b, c) lässt sich durch das aufgedruckte Gebet die Funktion der Abbildung als Andachtsbild gut erkennen, wie auch bei der des Innsbrucker Verlags Fischer (5 cm × 10,7 cm; Abb. 6d) oder des Nürnberger Verlags Franz Schemm (4,4 cm × 8,8 cm; Abb. 6e) bei motivgleichem Frontbild mit über der Kirche schwebenden Hl. Corona.

## Fazit für die Praxis

- Innerhalb der Medizin- und Urologiegeschichte hat sich in der Erinnerungskultur hagiographisches Wissen erhalten, das nicht nur in Bezeichnungen für Krankenhausstationen, die der Urologie gewidmet sind, seinen Ausdruck findet. Meist ist dies mit dem Hl. Liborius als Patron gegen Harnsteine verbunden.
- Seuchenheilige sind innerhalb der Urologie bis auf den Hl. Rochus zumeist in Vergessenheit geraten, insbesondere, da Syphilis und Geschlechtskrankheiten eher negativ konnotiert sind.
- Für die Hl. Corona finden sich nur für den Ort St. Corona am Wechsel Hinweise für eine Verehrung bei Seuchen.
- Für die Urologie bleibt sie unbekannt.

## Footnotes

1 Eine Dokumentation zu urologischen Patrozinien in Düsseldorf und Umgebung ist in Vorbereitung.

2 „Liborius, halt für uns an, auf dass nicht Gries und Nierenstein die Strafen uns’rer Sünden sei’n.“

3 „Nieren- und Blasensteine treibt man ab, wenn man Igelblut trocknet, pulverisiert und in Wasser eingibt.“ [18]

4 „Gegen den Stein im menschlichen Körper ist der Lapis nephriticus, in Silber oder Gold gefasst und am Halse getragen, ein treffliches Mittel, indem er den Stein im menschlichen Körper auflöst.“ [18]

5 „Lasse den Samen von Sauerampfer durch einen unschuldigen Knaben sammeln und trage denselben in einem Beutelchen auf dem bloßen Leibe.“ [18]

6 Nur in einer Quelle, die auf St. Corona am Wechsel zurückgeht, werden (Vieh‑)Seuchen genannt. Wesentliche Lexika wie Schmidt und Beitl [23] geben Seuchen als Patrozinium nicht an. Hierauf weist Graf und Männig hin [24]. Seuchen waren seit alters her wichtige Gefahren in bäuerlichen Gemeinschaften. Es ist nachvollziehbar, dass man sich an eine örtlich besonders verehrte Heilige (in St. Corona am Wechsel ist Corona die Kirchen- und Ortspatronin) mit Gebeten in ganz besonderen Anliegen wandte. Nachweis nur Schäfer [25].
